# ROS1 kinase inhibition reimagined: identifying repurposed drug via virtual screening and molecular dynamics simulations for cancer therapeutics

**DOI:** 10.3389/fchem.2024.1392650

**Published:** 2024-07-29

**Authors:** Mohammed Alrouji, Sabina Yasmin, Fahad A Alhumaydhi, Sharaf E. Sharaf, Moyad Shahwan, Anas Shamsi

**Affiliations:** ^1^ Department of Medical Laboratories, College of Applied Medical Sciences, Shaqra University, Shaqra, Saudi Arabia; ^2^ Department of Pharmaceutical Chemistry, College of Pharmacy, King Khalid University, Abha, Saudi Arabia; ^3^ Department of Medical Laboratories, College of Applied Medical Sciences, Qassim University, Buraydah, Saudi Arabia; ^4^ Pharmaceutical Sciences Department, College of Pharmacy, Umm Al-Qura University, Makkah, Saudi Arabia; ^5^ Department of Clinical Sciences, College of Pharmacy and Health Sciences, Ajman University, Ajman, United Arab Emirates; ^6^ Center of Medical and Bio-Allied Health Sciences Research (CMBHSR), Ajman University, Ajman, United Arab Emirates

**Keywords:** ROS proto-oncogene 1, Midostaurin, Alectinib, drug repurposing, virtual screening, molecular dynamics simulations, essential dynamics

## Abstract

Precision medicine has revolutionized modern cancer therapeutic management by targeting specific molecular aberrations responsible for the onset and progression of tumorigenesis. ROS proto-oncogene 1 (ROS1) is a receptor tyrosine kinase (RTK) that can induce tumorigenesis through various signaling pathways, such as cell proliferation, survival, migration, and metastasis. It has emerged as a promising therapeutic target in various cancer types. However, there is very limited availability of specific ROS1 inhibitors for therapeutic purposes. Exploring repurposed drugs for rapid and effective treatment is a useful approach. In this study, we utilized an integrated approach of virtual screening and molecular dynamics (MD) simulations of repurposing existing drugs for ROS1 kinase inhibition. Using a curated library of 3648 FDA-approved drugs, virtual screening identified drugs capable of binding to ROS1 kinase domain. The results unveil two hits, Midostaurin and Alectinib with favorable binding profiles and stable interactions with the active site residues of ROS1. These hits were subjected to stability assessment through all-atom MD simulations for 200 ns. MD results showed that Midostaurin and Alectinib were stable with ROS1. Taken together, the study showed a rational framework for the selection of repurposed Midostaurin and Alectinib with ROS1 inhibitory potential for therapeutic management after further validation.

## 1 Introduction

Cancer is affecting millions of individuals across the globe (Sung et al., 2021). Over the past few decades, cancer therapeutics has gained significant momentum, i.e., modern scientific approaches that understand the molecular mechanisms involved in tumorigenesis have transformed it and management paradigms (Wicha et al., 2006; Batlle and Wilkinson, 2012; Ho and Gorski, 2019). Precision medicine opened up a new avenue in cancer therapeutics, i.e., therapies are designed to target specific molecular irregularities responsible for the onset and progression of tumors (Diamandis et al., 2010; Nassar et al., 2020). The ROS proto-oncogene 1 (ROS1) is a receptor tyrosine kinase (RTK) belonging to the insulin receptor family. It was initially identified as a proto-oncogene having a transforming potential in lung cancer. *ROS1* gene fusions have been discovered in a diverse array of cancers, including non-small cell lung cancer (NSCLC) (Gainor and Shaw, 2013; Lin and Shaw, 2017), glioblastoma (Ou et al., 2012), cholangiocarcinoma (Lee et al., 2015), and others (Drilon et al., 2021). These fusions cause tumorigenesis by enhancing uncontrolled cell proliferation, survival, and migration (Yoshida et al., 2013; Tilak et al., 2021). An array of studies revealed the significance of ROS1 in cancer therapeutics (Azelby et al., 2021).

**FIGURE 1:**
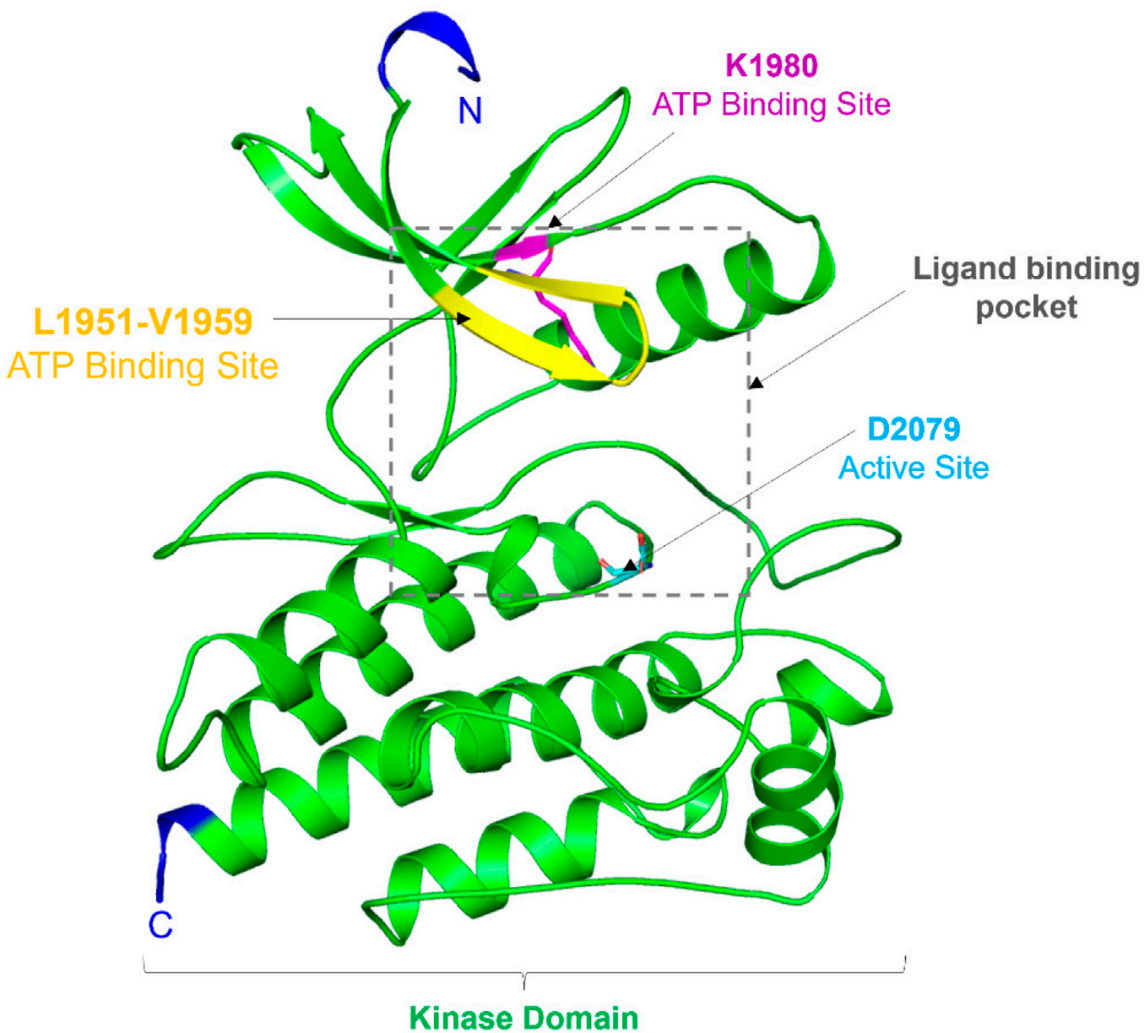
Structural topology of ROS1. Cartoon representation showed structural features of the ROS1 kinase domain. The figure was drawn in PyMOL using the PDB coordinates with ID: 3ZBF.

Structurally, the ROS1 protein consists of 2,347 amino acid residues with a molecular mass of 263,915 Da, featuring a kinase domain spanning 278 amino acid residues (from Leu1945 to Phe2222) (Parate et al., 2021). This kinase domain comprises an N-terminal β-strand domain and a C-terminal α-helical domain, connected by a hinge region crucial for ROS1’s catalytic activity. Notably, ROS1’s active site is characterized by Asp2079, while Lys1980 serves as the ATP binding site (Petrovic et al., 2022). While ROS1 shares structural similarities with typical protein kinases, its conformation around the active site exhibits distinctiveness. Comparing its structure to other kinases, the ROS1 gene demonstrates 49% sequence homology in the kinase domain and 77% identity at the ATP-binding site with ALK (Awad et al., 2013). Particularly, differences in ROS1, especially around the ATP binding site, hold significant implications for designing selective and potent competitive inhibitors. A visual representation of ROS1’s structural organization is depicted in Figure 1.

The development of potent small-molecule inhibitors has been instrumental in the success of cancer therapeutics (Hoelder et al., 2012; Millner and Strotman, 2016). Among the most well-known instances are Imatinib for BCR-ABL in chronic myeloid leukemia and Gefitinib for EGFR mutations in non-small cell lung cancer. (Bell et al., 2005). Crizotinib was the first-in-class inhibitor to gain FDA approval for ROS1-driven cancers, that show remarkable clinical efficacy (Yasuda et al., 2012). However, despite the early success of Crizotinib, the rise of resistance mutations has created considerable obstacles to its long-term effectiveness, forcing the investigation of alternate therapeutic approaches (Awad et al., 2013; Jiang et al., 2018). The reduced availability of specific ROS1 inhibitors for therapeutic purposes needs novel drug discovery approaches. This constraint has led to the use of medication repurposing methods to uncover novel therapeutic possibilities (Cha et al., 2018). Drug repurposing approach uses existing drugs to explore their potential for new therapeutic applications (Pushpakom et al., 2019) and is vital in a way that it effectively reduce the development timelines, and costs with established safety profiles (Park, 2019).

In the current study, we used virtual screening and molecular dynamics (MD) simulations to identify potential leads against ROS1. The study targets to find attractive options among FDA-approved medications as potential ROS1 inhibitors. Herein, we used a curated library of 3648 FDA-approved drugs from DrugBank (Wishart et al., 2018). The virtual screening approach includes systematically evaluating each compound’s capacity to interact with the ROS1 kinase domain. Through virtual screening, we identified potential drugs that could act as potent ROS1 inhibitors. Further, MD simulation deciphered the binding mechanisms and stability of the identified compounds within the ROS1 kinase domain. The study demonstrates the effectiveness of computational approaches in speeding up drug repurposing efforts thereby facilitating the identification of potential therapeutic alternatives for ROS1-driven cancers.

## 2 Materials and methods

### 2.1 Data collection and preparation

Bioinformatics programs such as MGL AutoDock Tools (Huey et al., 2012), AutoDock Vina (Trott and Olson, 2010), Discovery Studio Visualizer (Biovia, 2017), and GROMACS 2020 beta (Van Der Spoel et al., 2005) were utilized to conduct virtual screening and MD simulation investigations. Various online and standalone tools such as the RCSB Protein Data Bank (PDB) (Berman et al., 2000), DrugBank (Wishart et al., 2018), SwissPDB-Viewer (Kaplan and Littlejohn, 2001), and XMGrace (Turner, 2005) were used for data downloading and structure preprocessing. The crystal structure of human ROS1 with a resolution of 2.20 Å was downloaded from the PDB (Accession number: 3ZBF) for structure-based investigations in this study (Awad et al., 2013). The structure was examined and preprocessed using SwissPDB-Viewer and MGL tools for subsequent analyses. For the virtual screening phase, we curated a library of 3648 FDA-approved drugs, originally sourced from DrugBank. The 3D structures of we preprocessed and optimized the ligand structures using Open Babel (O'Boyle et al., 2011). The processed structural files of protein and ligands were utilized in subsequent structure-based molecular docking. The docking protocol underwent validation via a retrospective docking procedure, wherein the cocrystallized Crizotinib was redocked into the ROS1 binding site (Rampogu et al., 2019). The analysis indicated that Crizotinib, upon redocking, occupied the same position within the ROS1 binding site as in its co-crystallized state (Supplementary Figure S1). This observation between the docked and co-crystallized Crizotinib highlights the precision and reliability of our docking protocol, affirming its accuracy in aligning ligands within the ROS1 binding pocket.

### 2.2 Structure-based molecular docking

Molecular docking investigations were executed using the AutoDock Vina software, augmented by multiple Perl scripts. The preparation of the ROS1 crystal structure was conducted with meticulous attention to detail that compasses the addition of hydrogen atoms, charge assignments, and the assignment of appropriate atom types. The ROS1 protein’s binding site was delineated without prior knowledge to encompass the entire search space of the protein. Ligands were introduced into the ROS1 binding site freely employing a blind grid-based strategy. The dimensions of the grid were configured as 70 Å for the *X*-axis, 63 Å for the *Y*-axis, and 65 Å for the *Z*-axis, with a central reference point at coordinates of 7.961 for X, 37.925 for Y, and 6.669 for Z. The grid spacing was consistently set at 1.00 Å, while the exhaustiveness parameter was fine-tuned to a value of 8. These docking parameters were systematically optimized to broadly explore ligand conformations and orientations. For each ligand, individual docking simulations were conducted, and the resultant poses underwent ranking based on binding affinity scores and interaction energies. The molecular docking yielded binding affinity scores that were scrutinized to pinpoint potential candidates with high affinity for ROS1. Ligands were further sorted based on their docking scores. Detailed analysis of docked ligands’ interaction with important amino acids within the binding site was carried out to assess their consistency with known ROS1-ligand interactions.

### 2.3 Interaction analysis

Compounds exhibiting promising binding affinities with appropriate drug profiling were identified as candidates for further analysis. To explore potential binding modes for each compound, we employed the Vina Splitter program after a preliminary assessment of the docking data highlighted those with stronger binding affinities. These conformations were then examined comprehensively to assess their potential interactions with ROS1. This scrutiny was carried out using PyMOL and Discovery Studio Visualizer. Based on the interaction analysis, only compounds demonstrating specific interactions with ROS1 binding-site and active-site residues were chosen.

### 2.4 Biological property prediction and selection

We employed the PASS server (http://www.way2drug.com/passonline/predict.php) to predict and evaluate the biological properties of the selected compounds. These predictions were crucial in prioritizing compounds with favorable pharmacokinetic and safety profiles for further consideration (Lagunin et al., 2000). This tool harnesses the chemical structure of compounds to analyze their potential biological characteristics. It generates predictive scores for various biological attributes based on the “probability to be active (Pa)” and “probability to be inactive (Pi)” ratio. A higher Pa value indicates an increased likelihood of compounds possessing specific biological properties. The primary focus of this study was to identify biological indicators associated with ROS1 inhibitory properties. Consequently, the selected compounds from here were further investigated through MD simulations based on their promising predictions and potential as ROS1 inhibitors.

### 2.5 MD simulations

ROS1 was subjected to comprehensive all-atom MD simulations before and after binding with the identified compounds, Midostaurin and Alectinib, and the reference ROS1 inhibitor, Crizotinib. These simulations were carried out throughout 200 ns at a temperature of 300 K, employing the GROMOS 54A7 force field (Huang et al., 2011) within GROMACS 2020 beta (Van Der Spoel et al., 2005). Before initiating the MD simulations, meticulous preparation of the ROS1-ligand complexes was carried out. They were immersed in the SPC216 solvent model and enclosed within a periodic cubic box with a 1 nm edge distance. To maintain overall charge neutrality, an appropriate number of counterions were added. To resolve any potential steric clashes within the systems, energy minimization was conducted, involving 1500 steps of the steepest descent algorithm throughout 1000 picoseconds (ps). Before progressing to the production MD phase, preliminary steps were taken to ensure system relaxation and establish a stable starting point. This involved energy minimization and equilibration in NVT and NPT ensembles. Following a 1000-ps equilibration period under constant volume conditions and the imposition of periodic boundary constraints at a steady 1 bar pressure, the temperature of all systems gradually increased from 0 to 300 K. All four systems underwent a final MD run for 200 ns. The simulated trajectories were recorded at regular intervals of 2 femtoseconds for subsequent analysis. These resulting trajectories were thoroughly analyzed using GROMACS’ built-in tools and were visually represented using Visual Molecular Dynamics (VMD) (Humphrey et al., 1996) and XMGrace (Turner, 2005).

### 2.6 MD trajectory analysis

The resultant trajectories of MDS were examined for structural stability, conformational dynamics, and hydrogen bond interactions within ROS1 and the ROS1-ligand complexes. To assess the stability and fluctuations in specific regions of the complexes, we calculated the dynamics of root mean square deviation (RMSD), root mean square fluctuation (RMSF), radius of gyration (*Rg*), solvent accessibility surface area (SASA), and secondary structures elements. Further, hydrogen bonding patterns were explored to get insights into the binding mechanisms and critical interactions in ROS1 and the ROS1-ligand complexes. In addition, principal component analysis (PCA) and free energy landscape (FEL) techniques were performed to extract dominant conformational modes and identify representative structures within the complexes.

### 2.7 MMPBSA calculation

The Molecular Mechanics Poisson Boltzmann surface area (MMPBSA) is a useful approach to studying biomolecular interactions between protein and ligand (Kumari et al., 2014). Here, MD scripts were utilized to carry out the MM-PBSA calculations (Bhardwaj et al., 2021). The MM-PBSA analysis was conducted using a trimmed stable trajectory of 10 ns in the g_mmpbsa module available at https://rashmikumari.github.io/g_mmpbsa/(Baker et al., 2001). This tool utilizes the MM-PBSA approach to assess the binding energy components. The output, in the form of a DAT file, and analyzed by a Python script incorporated within the package. This script calculates the energetic contribution of each residue and the overall binding energy. The following equation is employed for the computation of the binding energy:

$${∆G}_{binding}=G_{complex}-\left(G_{receptor}+G_{ligand}\right)$$

where, ${∆G}_{binding}$ signifies the total binding energy of the protein-ligand complex, $G_{receptor}$ signifies the binding energy of protein and $G_{ligand}$ signifies the binding energy of the ligand.

## 3 Results and discussion

### 3.1 Molecular docking

Molecular docking screening was performed on a curated library of 3648 FDA-approved drugs to explore their binding potential towards ROS1. The screening process systematically evaluated each compound’s ability to interact with the ROS1 kinase domain’s active site to identify molecules with favorable binding profiles. The analysis showed that many compounds screened from the library displayed significant affinity for ROS1. The top 10 selected compounds showed appreciable binding potential with docking scores ranging from −11.5 to −10.3 kcal/mol (Table 1). The comparative analysis showed that all these molecules exhibited better docking scores than the reference inhibitor, Crizotinib. This indicates that the selected hits might have a high potential to bind ROS1 and inhibit its kinase activity. These compounds showed their potential to be explored for their drug profiling and molecular interactions with ROS1.

**TABLE 1:** The screened top 10 molecules and their docking parameters with ROS1.

S. No.	Drug	Binding free energy (kcal/mol)	Ligand efficiency (kcal/mol/non-H atom)	Torsional energy
1	Ergotamine	−11.5	0.2674	1.5565
2	Midostaurin	−11.2	0.2605	1.8678
3	Dihydroergocristine	−11.0	0.2444	1.8678
4	Bisdequalinium Chloride	−10.8	0.2455	0
5	Rifaximin	−10.8	0.1895	2.1791
6	Midostaurin	−10.8	0.2077	2.4904
7	Paritaprevir	−10.5	0.1909	2.1791
8	Alectinib	−10.3	0.2861	0.9339
9	Conivaptan	−10.3	0.2711	1.2452
10	Dutasteride	−10.3	0.2784	1.2452
11	Crizotinib	−8.6	0.2867	1.8678

### 3.2 Selection of promising candidates and pass analysis

The PASS is an internet-based tool designed to forecast numerous biological characteristics associated with a given compound, encompassing approximately 4,000 attributes (Lagunin et al., 2000). The PASS server was leveraged to assess the potential biological effects of the identified compounds. Based on the results of molecular docking screening, two compounds, Midostaurin and Alectinib emerged as highly promising candidates considering their drug profiles, structural characteristics, and predicted binding affinities. It was found that Midostaurin and Alectinib exhibited favorable drug profiling and PASS predictions. The PASS assessment revealed that both compounds exhibited anti-cancer attributes, aligning with our initial investigations. The compounds showcased kinase inhibitory and antineoplastic (including non-small cell lung cancer) potential possessing a Pa value exceeding 0.499 for Midostaurin and 0.205 for Alectinib, wherein Pa surpassed Pi (Table 2). Both Midostaurin and Alectinib exhibited promising predictions related to ROS1 inhibitory properties. The analysis suggested a high probability of these molecules with ROS1 inhibition potential that further supports their selection for interaction analysis.

**TABLE 2:** Selected compounds and their biological properties by Way2Drug PASS server.

Drug	Molecular strcuture	Iupac name	Pa	Pi	Biological activity
Midostaurin	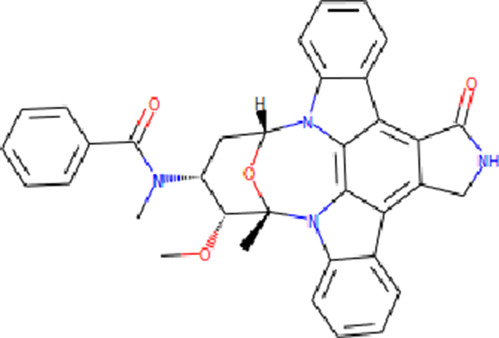	N-[(2S,3R,4R,6R)-3-methoxy-2-methyl-16-oxo-29-oxa-1,7,17-triazaoctacyclo[12.12.2.12,6.07,28.08,13.015,19.020,27.021,26]nonacosa-8,10,12,14,19,21,23,25,27-nonaen-4-yl]-N-methylbenzamide	0.961	0.004	Protein kinase inhibitor
			0.813	0.010	Antineoplastic
			0.499	0.006	Antineoplastic (non-small cell lung cancer)
Alectinib	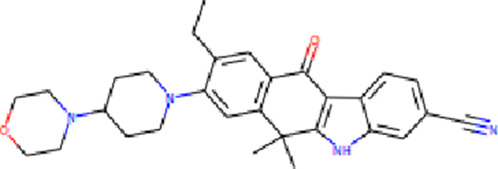	9-ethyl-6,6-dimethyl-8-(4-morpholin-4-ylpiperidin-1-yl)-11-oxo-5H-benzo [b]carbazole-3-carbonitrile	0.349	0.126	Antineoplastic
			0.218	0.060	Antineoplastic (non-small cell lung cancer)
			0.205	0.153	Antimetastatic
Crizotinib	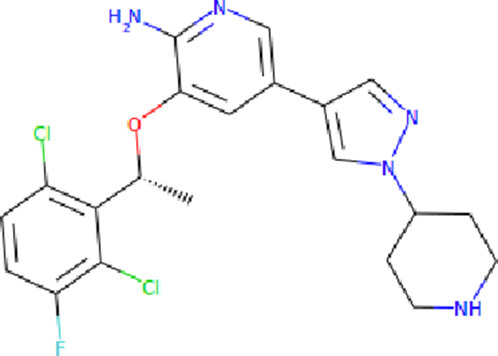	3-[(1R)-1-(2,6-dichloro-3-fluorophenyl)ethoxy]-5-(1-piperidin-4-ylpyrazol-4-yl)pyridin-2-amine	0.522	0.017	Protein kinase inhibitor
			0.399	0.104	Antineoplastic
			0.192	0.058	Tyrosine kinase inhibitor

These molecules were further evaluated for their pharmacokinetic properties. Pharmacokinetic properties play a pivotal role in shaping a compound’s absorption, distribution, metabolism, excretion, and toxicity profiles. The molecules chosen for analysis showcased promising ADMET profiles devoid of any concerning toxic patterns (Table 3). Notably, these compounds displayed a notable resemblance in their ADMET characteristics, validating their selection for further investigation. Midostaurin showed better pharmacokinetic properties than Crizotinib as it showed to be OCT2 substrate.

**TABLE 3:** Selected compounds and their pharmacokinetic properties by pkCSM server.

S. No.	Molecule	Absorption	Distribution	Metabolism	Excretion	Toxicity
		*GI Absorption (%)*	*BBB permeation*	*CYP2D6 Inhibitor*	*OCT2 substrate*	*AMES*
1	Midostaurin	98.086	−0.802	No	No	No
2	Alectinib	94.631	0.193	No	No	No
3	Crizotinib	92.006	−1.164	No	Yes	No

### 3.3 Binding modes and interactions

**FIGURE 2:**
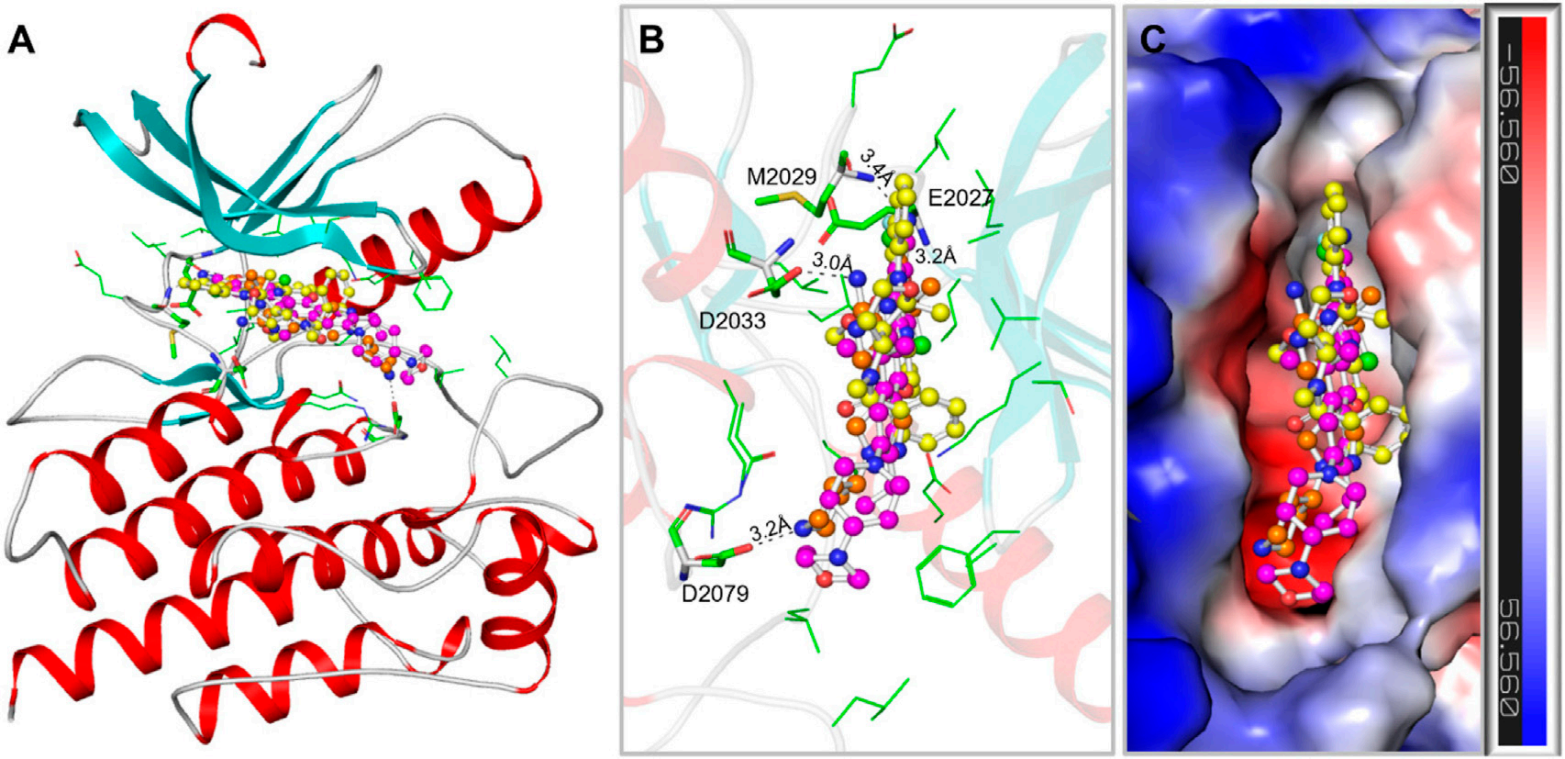
ROS1 in complex with the elucidated compounds. **(A)** ROS1 cartoon representation with Midostaurin (yellow), Alectinib (magenta), and Crizotinib (orange). **(B)** Magnified view of ROS1 interaction withMidostaurin, Alectinib, and Crizotinib. **(C)** Surface potential view of the ROS1 occupied with the elucidated compounds.

The binding prototype and interactions analysis provided insights into the binding modes and interactions between Midostaurin and Alectinib with ROS1. Both compounds displayed consistent interactions with important residues within the binding site, reinforcing their potential as ROS1 inhibitors (Figure 2). During the binding, Midostaurin and Alectinib formed hydrogen bonds with vital residues in the active site of ROS1, including Asp2079. These interactions stabilized the complex and suggested that Midostaurin and Alectinib effectively targeted the kinase domain (Figure 2A). Both compounds demonstrated strong interactions with the ROS1 kinase domain, primarily through multiple polar interactions with residues Glu2027, Met2029, Asp2033, and Asp2079 (Figure 2B). These residues make ROS1 binding pocket that plays an important role in its functional activity (Parate et al., 2021). Midostaurin forms one conventional hydrogen bond with Glu2027 with a distance of 3.2Å and one carbon-hydrogen bond along with several other close interactions. At the same time, Alectinib forms one conventional hydrogen bond with Met2029 with a distance of 3.4Å along with several other close interactions. Both compounds share similar interaction patterns as the co-crystalized reference ROS1 inhibitor, Crizotinib with a good complementarity fit (Figure 2C). These interactions along with several hydrophobic interactions contributed to the stability of the complex and highlighted Midostaurin and Alectinib’s potential as effective ROS1 inhibitors. Midostaurin and Alectinib were also docked with ROS1 homologous kinase ALK1 where they showed binding affinity of −10.2 and −10.4 kcal/mol, respectively (Supplementary Figure S2). While comparing, Midostaurin showed higher affinity towards ROS1 than ALK1, showing its preferential binding towards ROS1 kinase.

**FIGURE 3:**
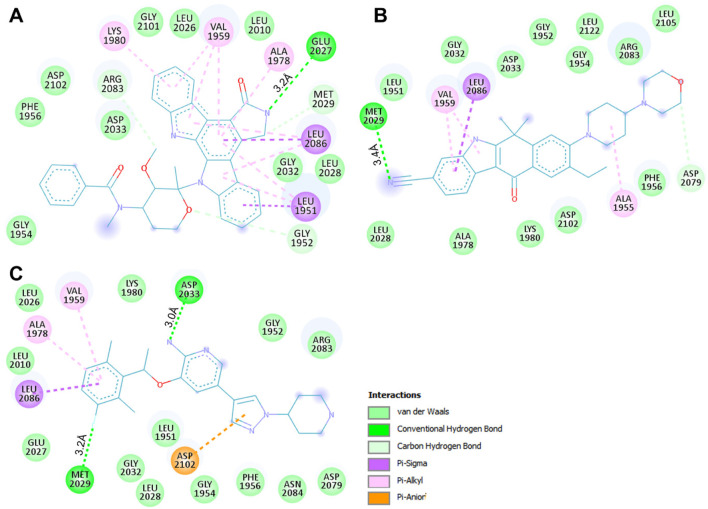
Interactive plots of ROS1 binding pocket residues with **(A)** Midostaurin, **(B)** Alectinib, and **(C)** Crizotinib.

We conducted an in-depth analysis to enhance our comprehension of the non-covalent interactions occurring between ROS1 and the identified compounds (Figure 3). This analysis holds significance in unveiling the nature and specific locations of intramolecular connections between the compounds and the protein. Notably, in the cases of Midostaurin and Alectinib, the interactions were further scrutinized through the generation of 2D plots, serving as visual aids to elucidate these interactions. The resulting 2D plots for both compounds are visually represented in Figures 3A,B. Midostaurin establishes a single conventional hydrogen bond with Glu2027 at a distance of 3.2Å, and it also forms a carbon-hydrogen bond, in addition to various other nearby interactions. Conversely, Alectinib creates one conventional hydrogen bond with Met2029, spanning a distance of 3.4Å, alongside several other proximal interactions. Interestingly, both compounds manifest a comparable binding pattern, suggesting a shared mechanism of action as the co-crystalized reference ROS1 inhibitor, Crizotinib (Figure 3C). The analysis unveiled that Midostaurin establishes close interactions with the ATP-binding site, primarily involving Lys1980, whereas Alectinib interacts with Asp2079 situated within the active site. These interactions, including a network of hydrophobic interactions, collectively underpinned the stability of the complex, underscoring the promising potential of Midostaurin and Alectinib as potent ROS1 inhibitors with significant therapeutic potential.

### 3.4 MD simulation

To gain deeper insights into the binding mechanisms, stability, and time-evolution dynamics of Midostaurin and Alectinib within the ROS1 kinase domain, we conducted all-atom MD simulations spanning 200 ns. The simulation analysis helps in understanding the dynamic behavior and stability of the drug-protein complexes.

#### 3.4.1 Structural deviation and compactness

**FIGURE 4:**
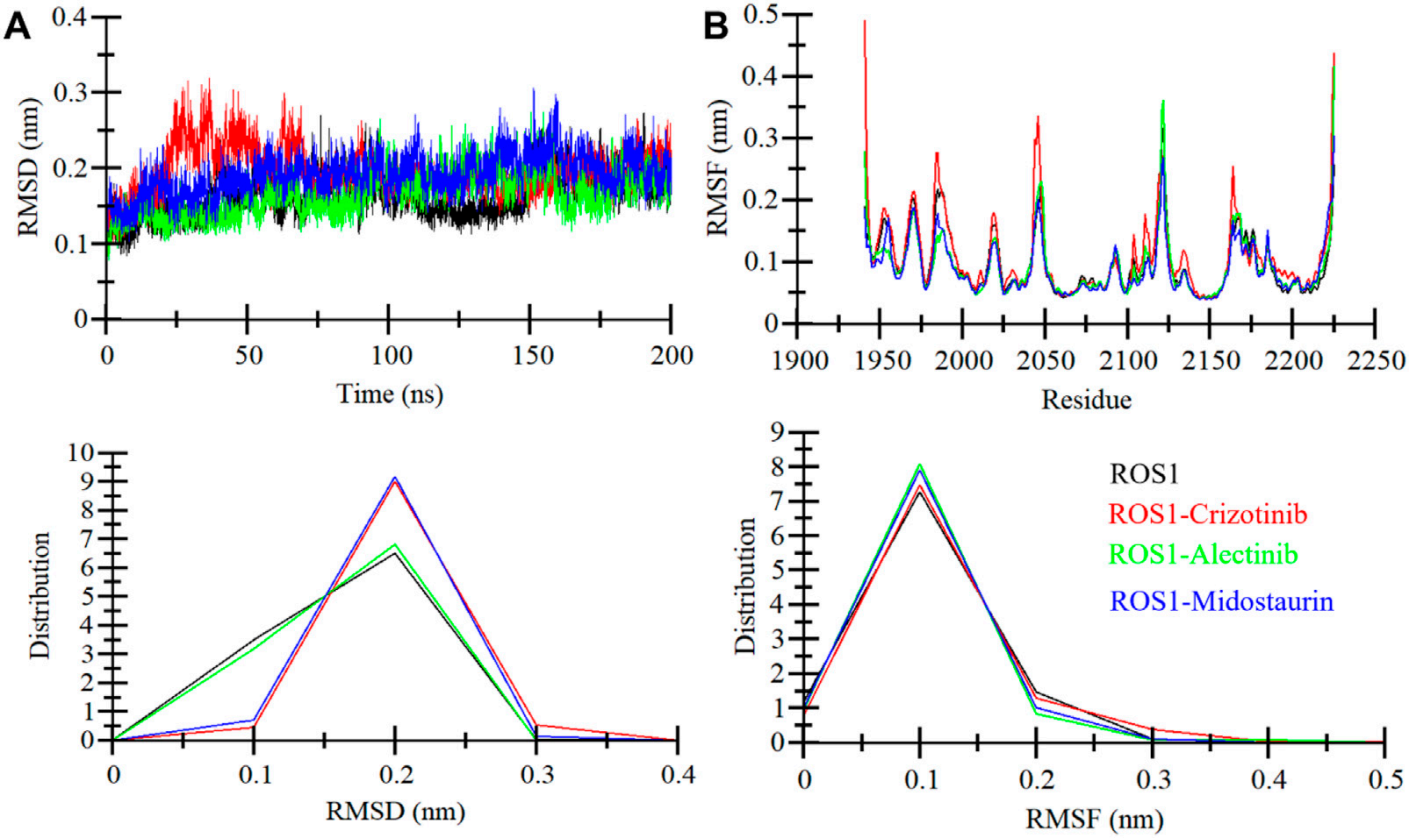
ROS1 dynamics with Crizotinib, Midostaurin, and Alectinib binding. **(A)** RMSD plot of ROS1 with Crizotinib, Midostaurin, and Alectinib. **(B)** Average residual fluctuations (RMSF) of ROS1 and its complexes with Crizotinib, Midostaurin, and Alectinib. The probability distribution function (PDF) is depicted in the lower panels.

When a ligand binds to the active site of a protein molecule, it can induce significant conformational alterations in its structure (Maruyama et al., 2023). These alterations in the protein’s conformation can exert a substantial influence on its biological activity and function (Pitera, 2014). To investigate the conformational changes in ROS1 before and after ligand binding, we examined RMSD of the simulated systems. The analysis focused on the time-evolution of the RMSD variations in the ROS1 protein before and after its interaction with Crizotinib, Midostaurin, and Alectinib (Figure 4A). The RMSD plot suggests that Midostaurin and Alectinib-bound states exhibit minor fluctuations compared to the ROS1-Crizotinib state. By evaluating RMSD values across all the systems, it was indicated that the simulation maintains equilibrium over the course of 200 ns, without any major conformational changes. Some slight RMSD fluctuations, approximately 0.15 nm (nm), are evident in the 20–70 ns region following Crizotinib binding (Figure 4A). To further assess stability, we examined the RMSD distribution plot, revealing a probability distribution function (PDF) centered primarily around 0.1–0.3 nm (Figure 4A, lower panel). This centered distribution strongly supports the system’s overall stability. In summary, the RMSD analysis underscores the stability of ligand-bound systems throughout the simulation. Interestingly, the ROS1-Alectinib and ROS1-Midostaurin complexes demonstrate greater stability compared to the ROS1-Crizotinib complex.

To delve deeper into the flexibility of each amino acid within ROS1, we generated and examined RMSFs. This metric serves as a valuable indicator of the lingering vibrations within the protein structure. The RMSF plot, depicted in Figure 4B, illustrates a consistent pattern across all four systems. These residual fluctuations within the protein exhibit stability and are notably reduced upon the binding of Midostaurin and Alectinib, compared to Crizotinib. This underscores the stability of the resulting complexes. Our analysis further revealed that the residues interacting with Midostaurin and Alectinib remained exceptionally stable throughout the study.

**FIGURE 5:**
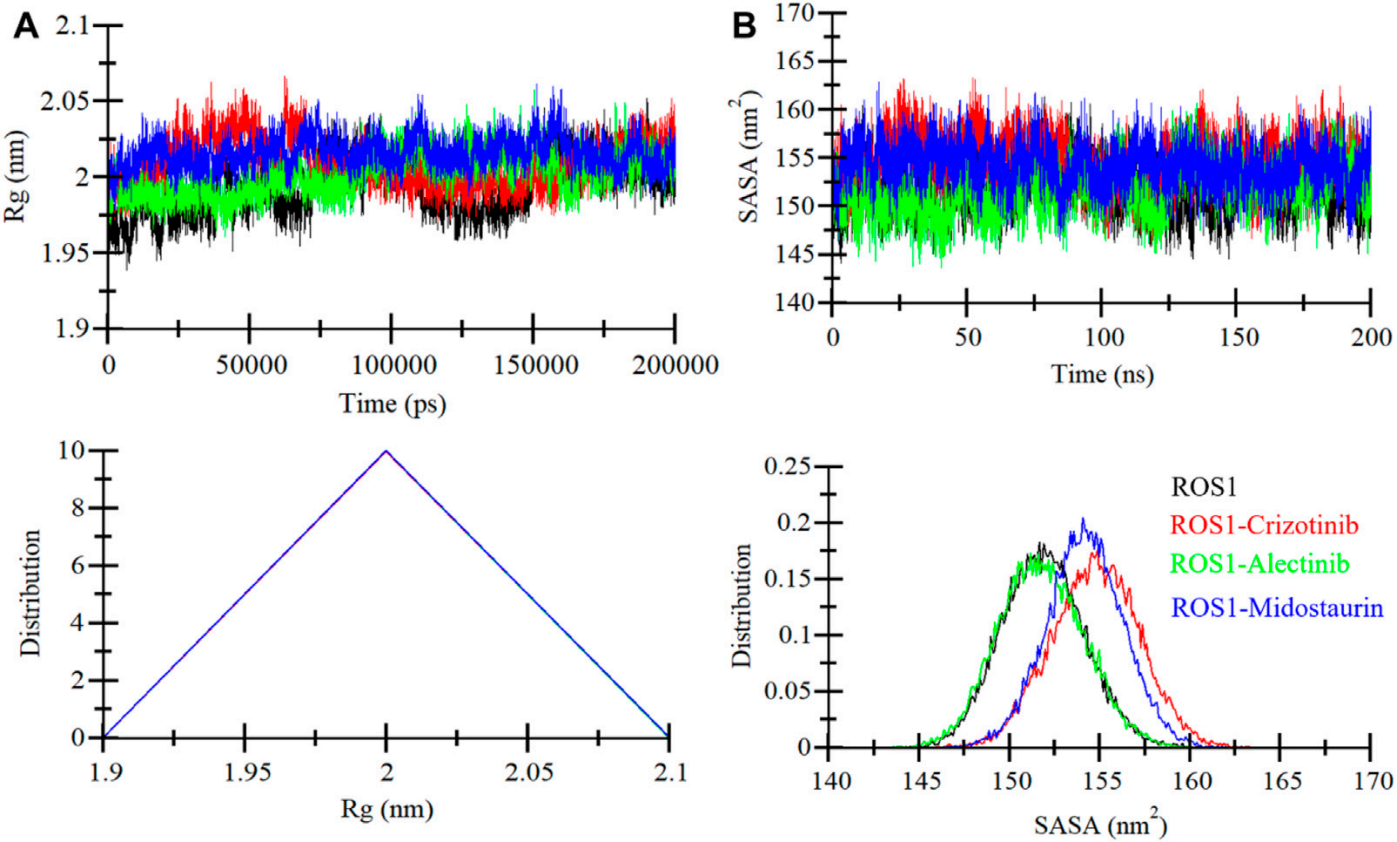
ROS1 structural compactness as a function of time. **(A)** The evolution of ROS1 Rg before and after binding to Crizotinib, Midostaurin, and Alectinib. **(B)** SASA plot of ROS1 before and after binding to Crizotinib, Midostaurin, and Alectinib. The PDFs are displayed in the lower panels.

*Rg* is a critical parameter that sheds light on the tertiary structure’s degree of compactness. In this investigation, we examined the time dependent *Rg* behavior of ROS1 to ascertain its compactness. The analysis contained time-evolution of *Rg* values for the ROS1-Midostaurin, ROS1-Alectinib, and ROS1-Crizotinib complexes (Figure 5A). The MD trajectories of these complexes displayed stable *Rg* values within the range of 1.95 nm–2.05 nm (Figure 5A, upper panel). The comparative analysis suggests that ROS1 maintains its structural stability and retains its folded state in the presence of both Midostaurin and Alectinib. This conclusion is further substantiated by the PDF plot, which indicates a uniform distribution of *Rg* values following the binding of Midostaurin and Alectinib to ROS1 (Figure 5A, lower panel).

The SASA serves as a crucial parameter that offers insights into a protein’s folding and stability (Zhu et al., 2002). It quantifies the area of a protein’s surface accessible to the surrounding solvent (Marsh and Teichmann, 2011). We evaluated the stability of ROS1 when interacting with Midostaurin and Alectinib based on their SASA values during the simulation (Figure 5B). The resulting plot displayed a consistent pattern, revealing minimal fluctuations in SASA throughout the simulation (Figure 5B, upper panel). These unchanging SASA values signify the robust stability of the protein-ligand complexes. Much like the *Rg* analysis, the SASA values remained uniform and exhibited no structural folding or compactness variations during the simulation. Further reinforcement of this stability trend was observed in the PDF plot for the ROS1 complexes with Alectinib, Midostaurin, and Crizotinib demonstrating a similar stabilizing effect on the protein (Figure 5B, lower panel).

#### 3.4.2 Hydrogen bond analysis

**FIGURE 6:**
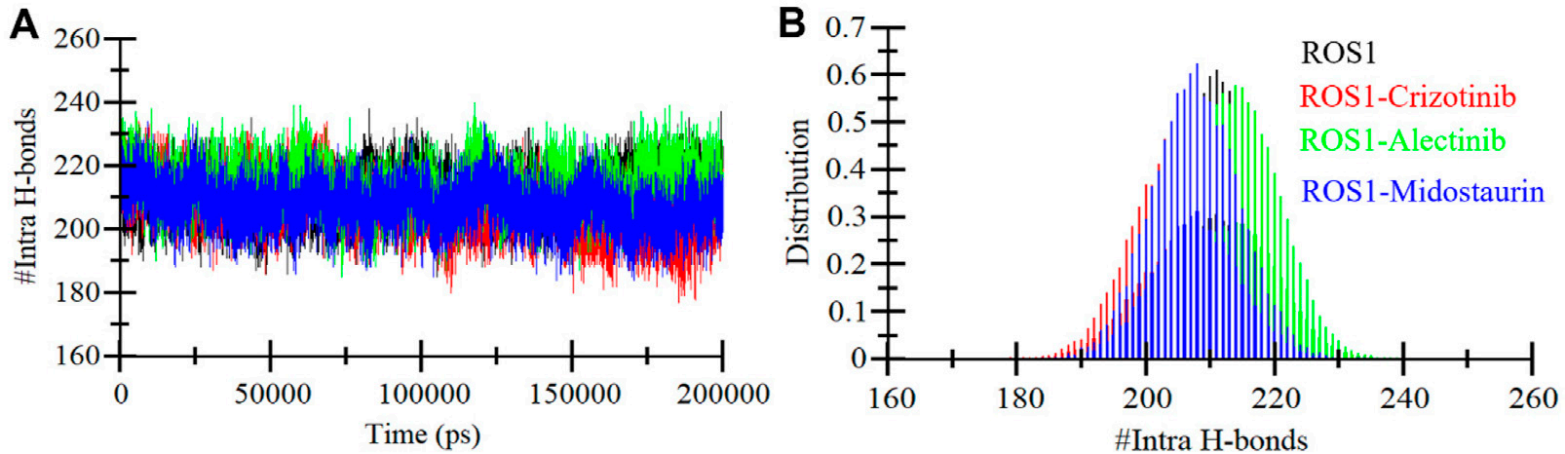
Intramolecular hydrogen bonding. **(A)** Time evolution of intra-ROS1 hydrogen bonds before and after Crizotinib, Alectinib, and Midostaurin binding. **(B)** The PDF of intramolecular hydrogen bonds in ROS1.

Ensuring the stability of the protein structure relies significantly on intramolecular hydrogen bonding (Menéndez et al., 2016). Analyzing hydrogen bonds can provide valuable insights into the protein’s conformational changes and compactness. We evaluated the formation of intramolecular hydrogen bonds within ROS1 using the MD simulations trajectories of both before and after ligand binding. The results are visually represented in Figure 6, illustrating the fluctuations in intramolecular hydrogen bonding formation with Crizotinib, Midostaurin, and Alectinib in ROS1. Notably, the plot reveals that even upon binding with Midostaurin and Alectinib, the formation of intramolecular hydrogen bonds within ROS1 remains continuous (Figure 6A). Furthermore, the PDF of hydrogen bonding displays a consistent pattern across all four systems, highlighting the stability of intramolecular hydrogen bonding (Figure 6B).

**FIGURE 7:**
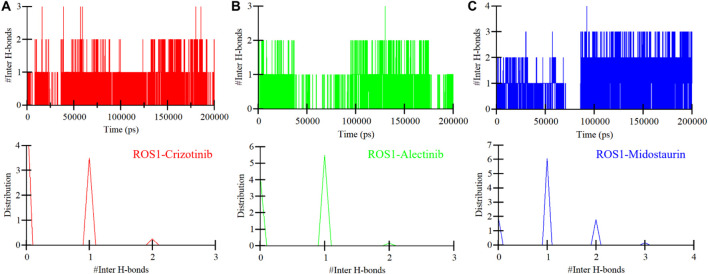
Intermolecular hydrogen bonding in ROS1 and **(A)** Crizotinib, **(B)** Alectinib, and **(C)** Midostaurin as a time function. The PDF of the data distribution is in the lower panels.

To further study the stability of polar interactions between ROS1 and Midostaurin, as well as Alectinib, we evaluated the intermolecular hydrogen bonds. These bonds’ directionality and specificity hold significant importance in comprehending protein kinetics (Yunta, 2017). In the ROS1-Crizotinib complex, we observed the formation of an average of one hydrogen bond, occasionally increasing to three, as depicted in the upper panel of Figure 7A. At the same time, the ROS1-Midostaurin complex also exhibited an average of one hydrogen bond, occasionally reaching 3 bonds (Figure 7B). Conversely, the ROS1-Alectinib complex exhibited an average of two hydrogen bonds, occasionally reaching four bonds (Figure 7C). The analysis presented in the PDF plots demonstrated a consistent distribution of intermolecular hydrogen bonds, with higher occurrences of one hydrogen bond in three complexes (Figure 7, lower panels). These results indicate that the binding of Midostaurin and Alectinib to ROS1 is upheld by intermolecular hydrogen bonds, effectively preventing the ligands from dissociating.

#### 3.4.3 Secondary structure dynamics

**FIGURE 8:**
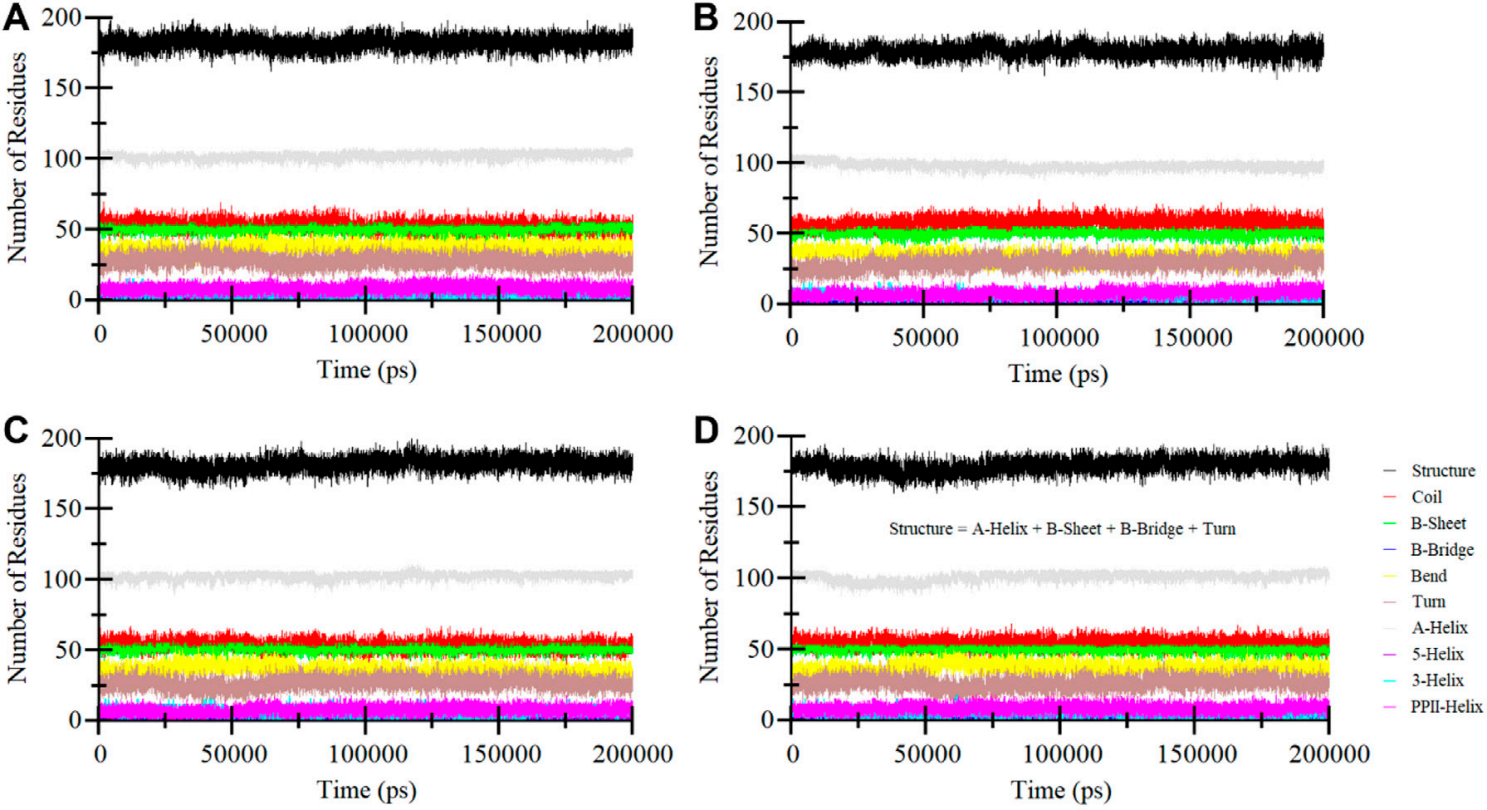
Secondary structure elements dynamics in **(A)** ROS1, **(B)** ROS1-Crizotinib, **(C)** ROS1-Midostaurin, and **(D)** ROS1-Alectinib.

Secondary structure elements play a critical role in maintaining the overall structural conformation of a protein. We conducted an in-depth exploration of the temporal changes in the secondary structure elements to evaluate the impact of Midostaurin, and Alectinib binding on ROS1 (Figure 8). The analysis revealed that the secondary structure composition of ROS1 remained remarkably consistent throughout the simulation (Figure 8A). Simultaneously, upon binding of Crizotinib, Midostaurin, and Alectinib, the secondary structure makeup of ROS1 also displayed a high degree of stability (Figures 8B–D). At some time points, the alterations in the secondary structure of ROS1 following binding with Midostaurin and Alectinib were relatively minor. This analysis highlighted the robustness and persistence of the secondary structure of ROS1 in the presence of Midostaurin and Alectinib. The preservation of these secondary structural elements serves to underline the stability of the native protein conformation in the presence of Midostaurin and Alectinib.

#### 3.4.4 Principal component analysis

**FIGURE 9:**
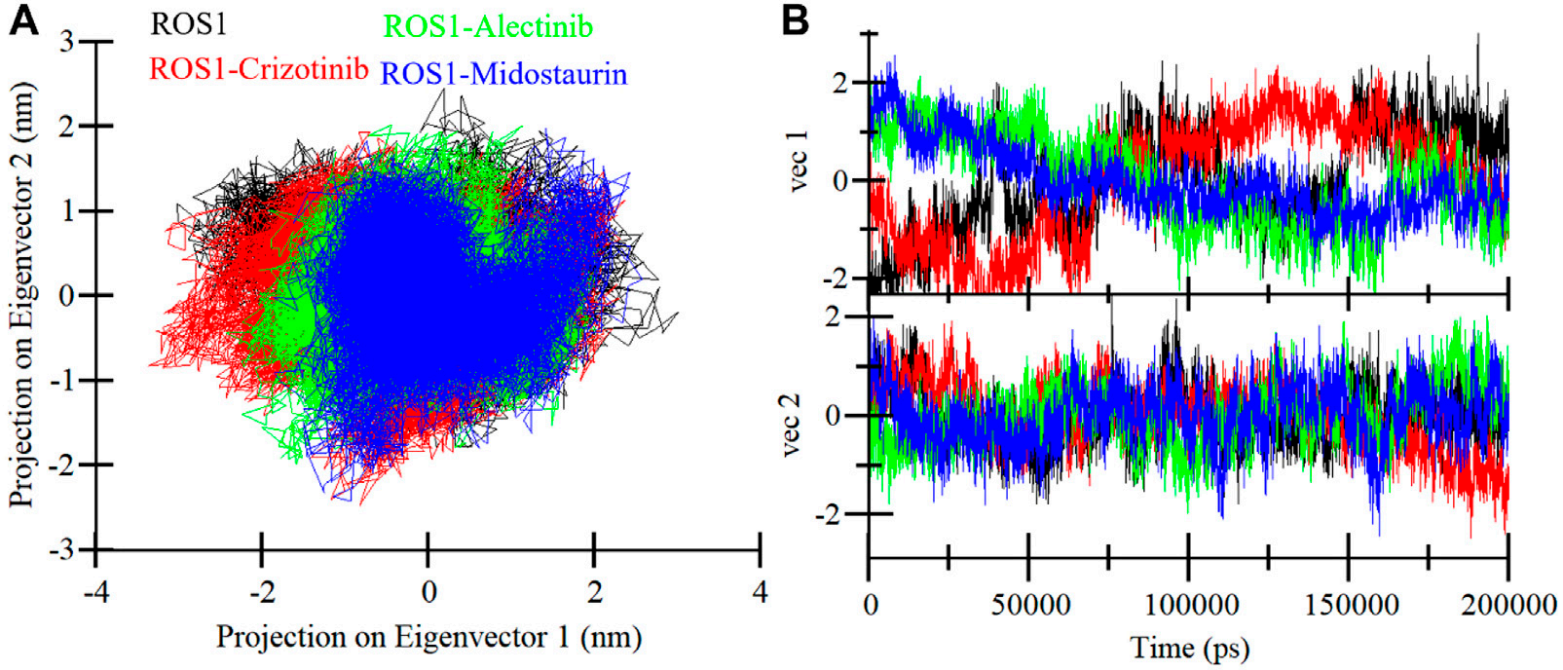
ROS1 conformational sampling in the PCA. **(A)** ROS1, ROS1-Crizotinib, ROS1-Midostaurin, and ROS1-Alectinib with 2D projection of conformational sampling. **(B)** The time-evolution of EVs plot for ROS1 and ROS1-ligand complexes.

The PCA is an invaluable technique used to get insights into the conformational exploration of a protein’s structure (Stein et al., 2006). In this investigation, we harnessed PCA to delve into the conformational dynamics of ROS1 and its complexes with Midostaurin, Alectinib, and Crizotinib. The results in a 2D map with two different Eigenvectors (EVs), EV1 and Ev2 showcase the conformational landscape of the three complexes (Figure 9). Notably, the ROS1-Midostaurin and ROS1-Alectinib complexes predominantly occupy a substantial portion of the same essential subspace as ROS1 in their unbound and co-crystalized states (Figure 9A). The time-evolution of EVs plot further revealed that ROS1 and ROS1-ligand complexes share almost the same phase, especially on EV2 (Figure 9B). The average traced for ROS1-Midostaurin, ROS1-Alectinib, and ROS1-Crizotinib complexes was found to be −5.17, 1.49, and −1.51 on EV1 and −2.59, −2.85, and 9.48 on EV2, respectively. Overall, these results indicate that the binding of the ligands does not exert a substantial influence on the protein’s conformational exploration.

### 3.5 Free energy landscapes

**FIGURE 10:**
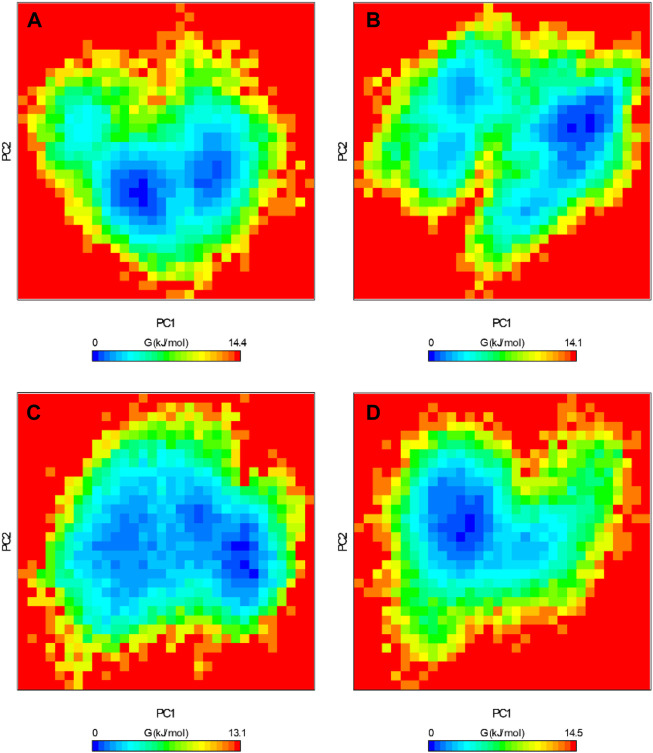
Free energy landscapes of **(A)** ROS1, **(B)** ROS1-Crizotinib, **(C)** ROS1-Midostaurin, and **(D)** ROS1-Alectinib

The FEL serves as a graphical representation of the folding process of a protein structure (Papaleo et al., 2009). It stands as a valuable tool for assessing the stability of proteins and protein-ligand complexes during MD simulations. We extracted the energy minima and conformational profiles of ROS1, ROS1-Crizotinib, ROS1-Midostaurin, and ROS1-Alectinib complexes while utilizing the first two principal components (PCs), PC1 and PC2. In the FEL plots, the deeper blue regions denoting lower energy levels closely approximate the native state of the protein (Figure 10). The FEL plot demonstrates that ROS1 primarily occupies 2 global minima, extending to encompass 2 basins (Figure 10A). The analysis indicates alterations in the size and location of confined phases containing 1-2 global minima upon binding Midostaurin and Alectinib to ROS1. At the same time, ROS1-Crizotinib, ROS1-Midostaurin, and ROS1-Alectinib are confined to a singular global minimum characterized by 1–2 basins (Figures 10B–D). Overall, FEL analysis indicated that ROS1 reached stable conformations even in the presence of bound ligands.

#### 3.5.1 MMPBSA analysis

The binding free energy for the ROS1-Crizotinib, ROS1-Midostaurin, and ROS1-Alectinib complexes was determined through MM-PBSA calculations. Binding energy represents the energy released during bond formation or the interaction between a ligand and protein (Bhardwaj et al., 2021). Better ligand-protein binding is reflected in lower binding energy values, with contributions from electrostatic, polar solvation, van der Waals, and SASA energies. Table 4 presents the average free binding energy values alongside their respective standard deviations. The results demonstrate that all energy components positively influenced the interactions of ROS1-Crizotinib, ROS1-Midostaurin, and ROS1-Alectinib. Specifically, ROS1-Midostaurin exhibited a more stable complex formation compared to ROS1-Crizotinib and ROS1-Alectinib.

**TABLE 4:** Different parameters of binding free energy for ROS1-ligand complexes calculated using MM-PBSA calculations of approach.

Complex	Δ*E*_Binding_ (kJ/mol)	SASA (kJ/mol)	Δ*E*_Polar solvation_ (kJ/mol)	Δ*E*_Electrostatic_ (kJ/mol)	Δ*E*_van der Waal_ (kJ/mol)
ROS1-Crizotinib	−106.882 ± 14.2	−14.05 ± 2.1	38.35 ± 10.3	−6.12 ± 1.9	−123.28 ± 10.04
ROS1-Midostaurin	−114.26 ± 10-.9	−16.94 ± 1.4	46.97 ± 7.9	−8.46 ± 2.7	−140.12 ± 8.2
ROS1-Alectinib	−103.54 ± 12.88	−15.28 ± 1.9	40.72 ± 10.1	−10.24 ±3.1	−134.76 ± 9.6

## 4 Conclusion

This study presents a comprehensive approach to addressing the therapeutic challenges posed by ROS1-driven cancers through drug repurposing. The study leveraged a combination of structure-based virtual screening and MD simulations to identify promising repurposed drugs capable of inhibiting ROS1 activity. The docking screening revealed a subset of drugs that exhibited favorable binding profiles and interactions within the ROS1 kinase domain. Two repurposed drugs, Midostaurin and Alectinib emerged as highly promising candidates based on their drug profiles, structural characteristics, and predicted binding affinities. Both compounds exhibited a high probability of possessing specific biological attributes associated with ROS1 inhibition in PASS analysis. The all-atom MD simulations confirmed that both compounds formed stable complexes with ROS1. Overall, the outcomes from the study lay the groundwork for future investigations. Rational design strategies can be employed to enhance their efficacy and minimize off-target effects of the elucidated molecules by exploiting their structural features. Structural modifications such as functional group substitutions, conformational changes, or the addition of moieties targeting specific ROS1 residues could lead to improved drug candidates. Further preclinical and clinical studies are required to evaluate the efficacy of Midostaurin and Alectinib as ROS1 inhibitors *in vitro* and *in vivo*. In conclusion, this study is a significant step forward in the pursuit of effective therapies for ROS1-driven cancers. Acknowledging study limitations, particularly predictive models’ constraints, emphasizes the need for experimental validation. Optimizing identified compounds for clinical translation demands further refinement, including structural modifications, pharmacokinetic, toxicity studies, and progression to preclinical and clinical trials.

## Data Availability

The original contributions presented in the study are included in the article/Supplementary Material, further inquiries can be directed to the corresponding author.
