# Supplementary material for: ROS1 kinase inhibition reimagined: identifying repurposed drug via virtual screening and molecular dynamics simulations for cancer therapeutics
Source: Front Chem. 2024 Jul 29;12:1392650. doi: 10.3389/fchem.2024.1392650 (PMC11317403; doi:10.3389/fchem.2024.1392650)
Supplement: Supplementary file 1 [file Presentation1.PPTX]

## Slide 1
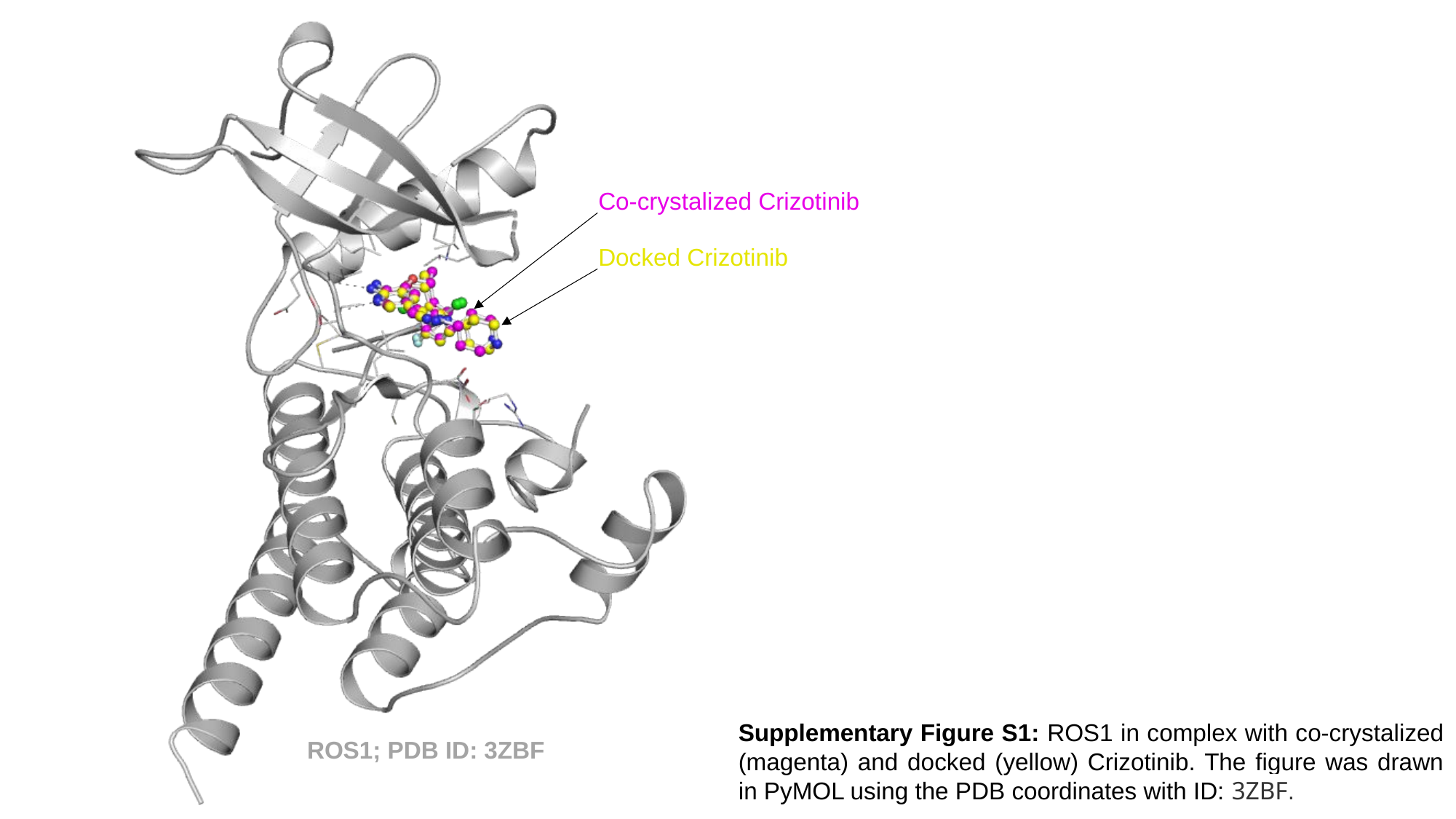

Co-crystalized Crizotinib
Docked Crizotinib
Supplementary Figure S1: ROS1 in complex with co-crystalized (magenta) and docked (yellow) Crizotinib. The figure was drawn in PyMOL using the PDB coordinates with ID: 3ZBF.
ROS1; PDB ID: 3ZBF

## Slide 2
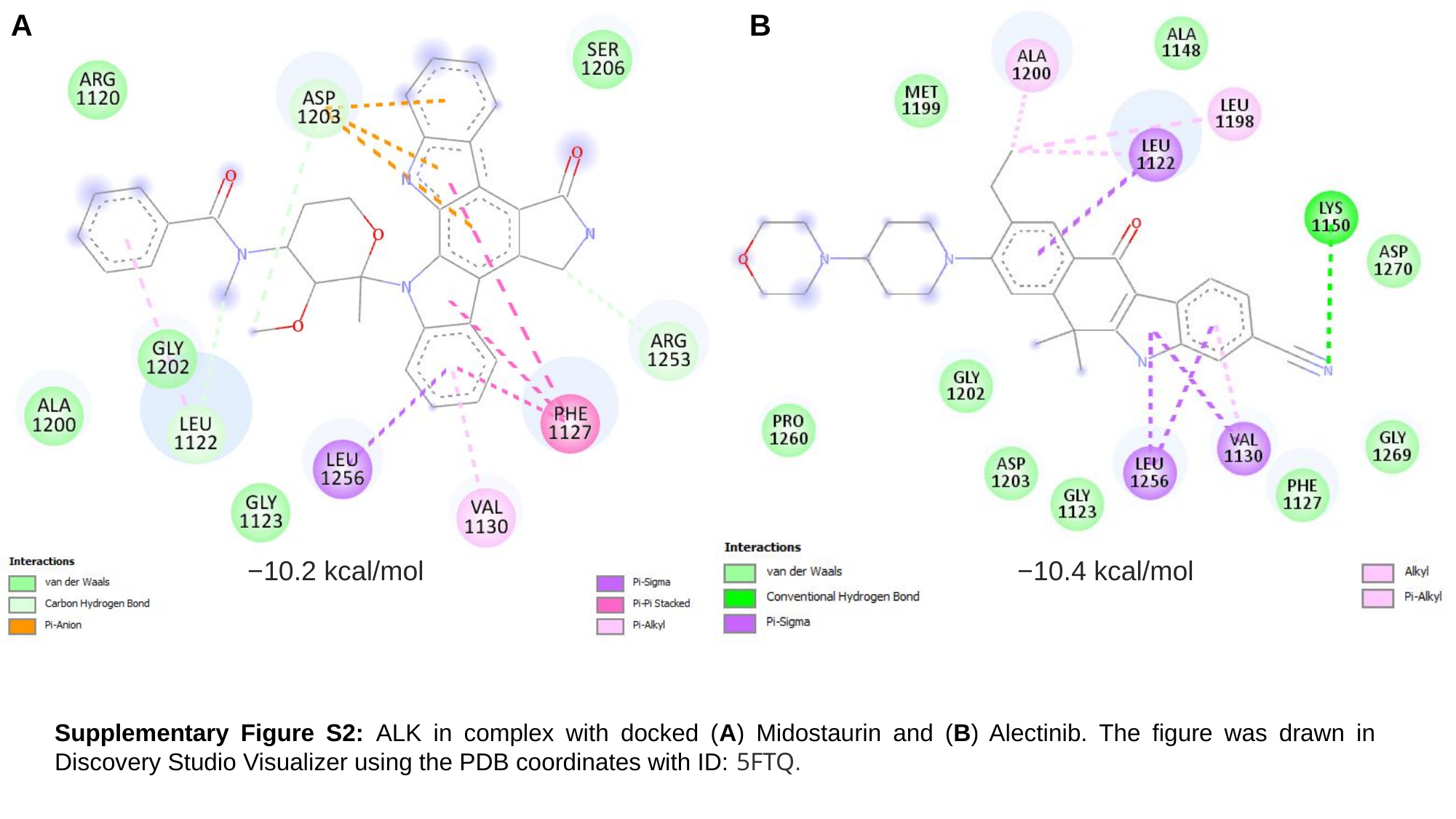

A
B
−10.2 kcal/mol
−10.4 kcal/mol
Supplementary Figure S2: ALK in complex with docked (A) Midostaurin and (B) Alectinib. The figure was drawn in Discovery Studio Visualizer using the PDB coordinates with ID: 5FTQ.

## Slide 3
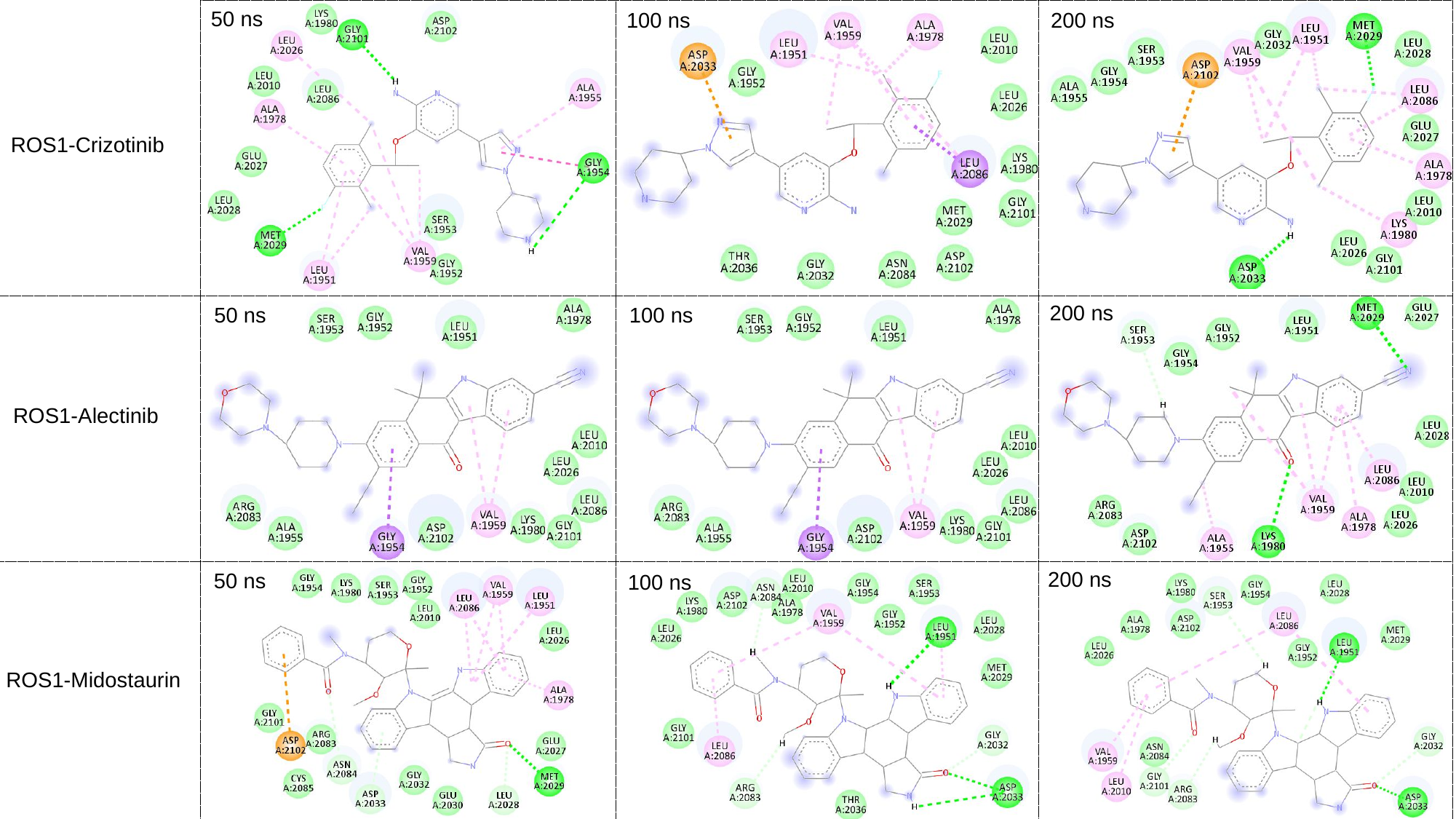

50 ns
100 ns
200 ns
ROS1-Crizotinib
200 ns
50 ns
100 ns
ROS1-Alectinib
200 ns
50 ns
100 ns
ROS1-Midostaurin
